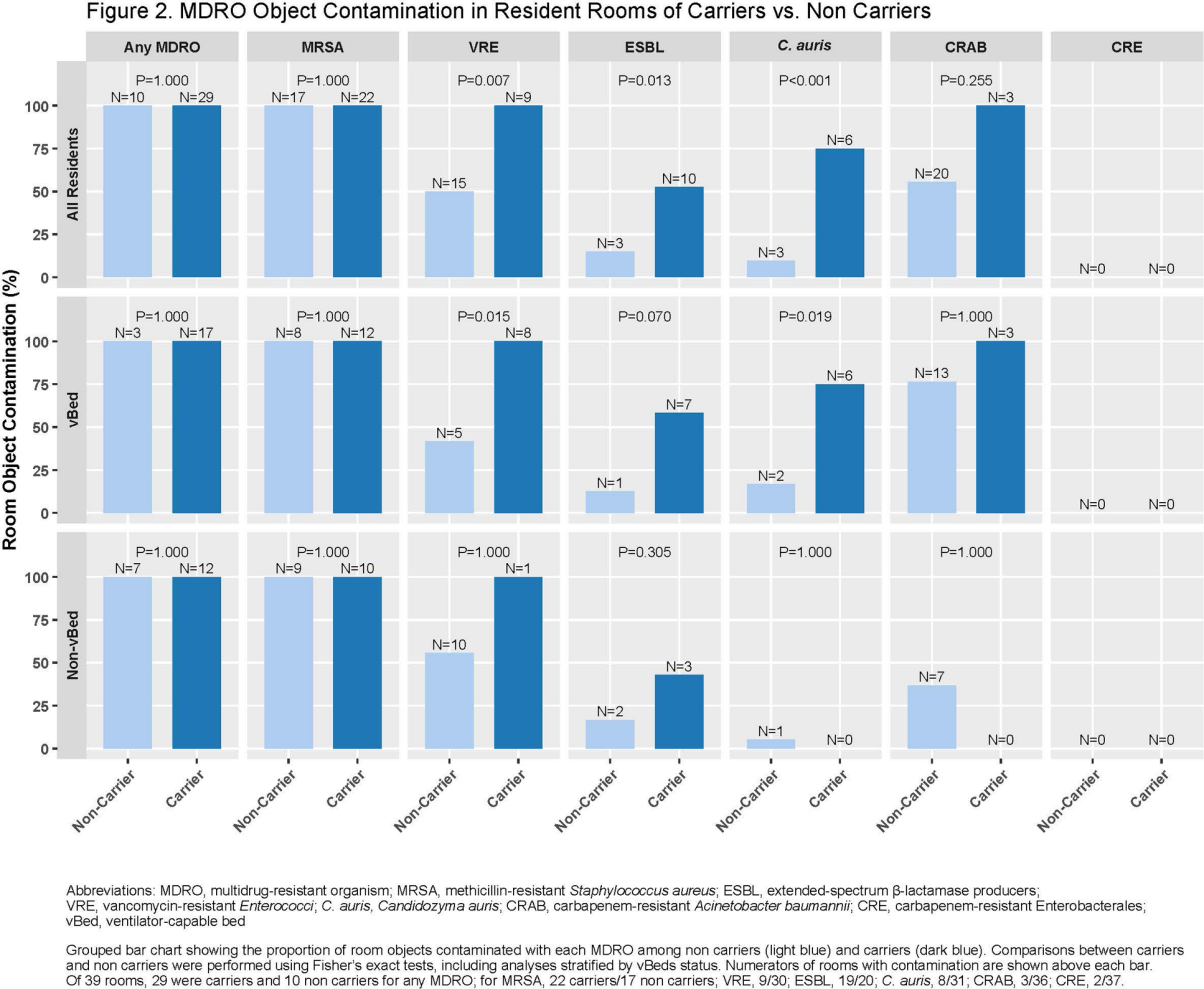# 240 Attributing Broad-Spectrum Antibiotic Use After Pediatric Solid Organ Transplantation to Culture Evaluation, Surgical Infection, and Re

**DOI:** 10.1017/ash.2026.10447

**Published:** 2026-06-23

**Authors:** Gabrielle Gussin, Raveena D. Singh, Raheeb Saavedra, Valeria Zaragoza, Jonathan Calzada, Alice Lee, Matthew San Pedro, Jahan Hosseinian, Gabriel Gadia, Julie Shimabukuro, Courtney Myers, Ayesha Khan, Cassiana E. Bittencourt, Susan Huang

**Affiliations:** 1 University of California, Irvine; 2 University of California, Irvine School of Medicine; 3 University of California Irvine School of Medicine; 4 UC Irvine; 5 University of California Irvine Health; 6 UCI

## Abstract

**Background:** vSNFs are known for exceedingly high MDRO prevalence, raising questions about the role of environmental contamination and persistence in this healthcare setting. **Methods:** Environmental sampling was conducted May-June 2025 in two vSNFs. High-touch objects in common areas (mobile/shower equipment, staff breakrooms, dining rooms, rehabilitation gyms) and resident rooms were swabbed using spongesicles, homogenized in trypticase soy broth, incubated 18-24 hours, and cultured for MRSA, VRE, ESBL, C. auris, CRAB, and CRE. On the same day, resident carriage of the same organisms was assessed at nares, hands, axilla/groin, and peri?rectal sites. All ventilator-capable (vBed) and non-vBed occupied beds were sampled except for one facility’s non-vBeds, which randomly sampled 50 beds due to size. The proportion of positive common area objects was compared with MDRO carriage prevalence using two-sample tests of proportions. Within resident rooms, object contamination was compared among carriers and non-carriers for each pathogen, overall and stratified by vBed status, using Fisher’s exact tests. **Results:** Sampling included 96 common area objects and 245 objects from 39 resident rooms (20 vBed, 19 non-vBed). MDRO contamination of high-touch objects in common areas was extensive (73.9%), including 77.8% of mobile equipment and 58.3% of common area objects (37.5% staff breakroom, 87.5% rehabilitation gym, 50.0% dining objects). The most frequently detected MDROs on objects were MRSA>CRAB>VRE>ESBL>C. auris>CRE, which differed from the sequence of MDRO carriage among residents: MRSA>ESBL>VRE>C. auris>CRAB>CRE. Object contamination and resident carriage were similar for MRSA (65.6% vs. 60.5%, p=0.43), VRE (19.8% vs. 23.3%, p=0.54), and CRE (1.0% vs. 4.1%, p=0.27). In contrast, contamination was markedly lower than carriage for ESBL (8.3% vs. 43.6%, p<0.001) and C. auris (3.1% vs. 25.6%, p<0.001), while CRAB contamination far exceeded carriage (21.9% vs. 11.0%, p=0.02) (Figure 1). Among bedrooms, all sampled rooms had some MDRO contamination, including all rooms of non-carriers. MRSA contamination was similar in the rooms of carriers and non-carriers; all other pathogens, contamination was substantially greater in rooms of carriers (Figure 2). **Conclusions:** The relationship between MDRO contamination and resident carriage in vSNFs showed clear pathogen?specific patterns. MRSA and VRE contamination closely mirrored resident carriage, whereas ESBL and C. auris were less frequent on surfaces despite high carriage. Notably, CRAB was disproportionately common in the environment, suggesting environmental persistence. Bedrooms of carriers had higher contamination, yet MDROs were also present in non-carrier rooms, contributing to ongoing transmission risk. Effective containment likely requires universal strategies supplemented by targeted, pathogen-specific efforts.

**Figure:**
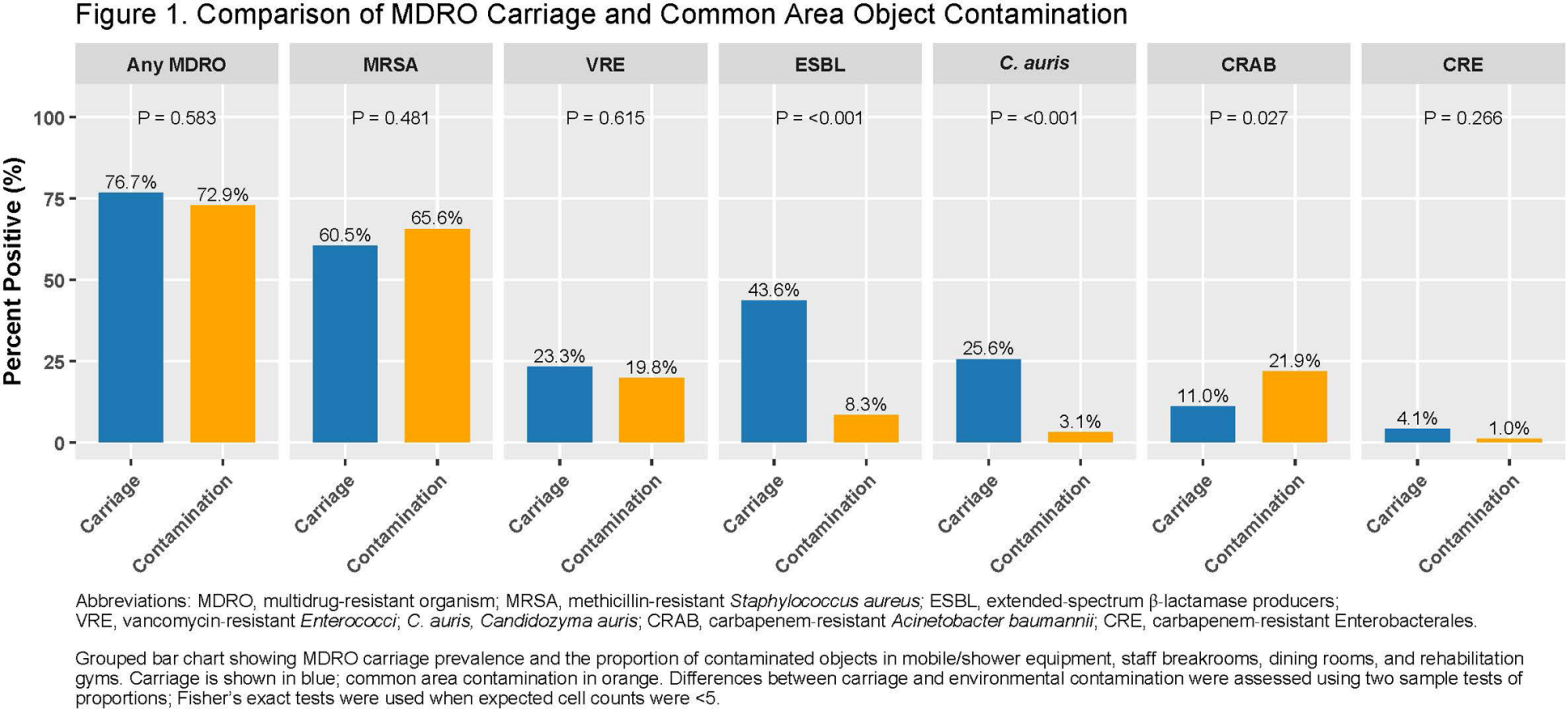

**Figure:**